# Data-Driven Prediction of Ammonia and Methane Concentrations and Emissions in Dairy Barns Using Artificial Neural Networks

**DOI:** 10.3390/ani16050824

**Published:** 2026-03-06

**Authors:** Luciano Manuel Santoro, Provvidenza Rita D’Urso, Claudia Arcidiacono, Salvatore Coco

**Affiliations:** 1 Building and Land Engineering Section, Department of Agriculture, Food and Environment (Di3A), University of Catania, 95123 Catania, Italy; luciano.santoro@phd.unict.it (L.M.S.);; 2 Section of Electrical, Electronics and Systems Engineering, Department of Electrical, Electronics and Informatics Engineering (DIEEI), University of Catania, 95123 Catania, Italy; salvatore.coco@dieei.unict.it

**Keywords:** livestock production, machine learning, dairy, climatic variables, diet, animal activity, ammonia, methane, artificial neural network

## Abstract

Ammonia (NH_3_) and methane (CH_4_) emissions from dairy farming pose significant environmental and climatic concerns. Several factors influence these emissions, including housing systems, diet, environmental conditions, and animal activity. Previous studies have mostly applied classical statistical methods to analyse the effect of these variables on gas emissions. Recent applications of artificial neural networks (ANNs) have not yet incorporated animal activity and diet as input variables. This study assessed the influence of climatic variables, animal activity and dietary intake on NH_3_ and CH_4_ concentrations and emissions from a naturally ventilated dairy barn under a Mediterranean climate. Multilayer perceptron (MLP) models were applied using environmental, activity, and dietary inputs. Model performance was evaluated using R, R^2^, MAE, MSE, SD, and RMSE. The results demonstrate that MLP models achieved accurate predictions, with R^2^ values of 0.93 and 0.96 for NH_3_ and CH_4_, respectively. Predictions incorporating climatic, diet and activity variables achieved the best performance. These findings suggest that ANN models, integrating these variables, represent effective tools for emission prediction, contributing to improved environmental management in dairy farming.

## Simple Summary

Dairy farming contributes to the emission of gases such as ammonia (NH_3_) and methane (CH_4_), which negatively impact the environment. NH_3_ causes water and soil pollution, while CH_4_ is a powerful greenhouse gas driving climate change. Direct measurement of these gases on farms is expensive and requires specialised equipment, limiting routine monitoring. This study applies artificial intelligence (AI) techniques to predict gas emissions from a dairy barn. AI models can learn complex patterns within data, enabling accurate prediction and reducing the need for continuous measurements. Moreover, the study identifies the main influencing variables (such as animal activity, climatic conditions, and dietary data) that significantly improve the accuracy of gas emission prediction. This approach is a cost-effective tool to support farmers in monitoring, achieving improved management decisions, and reducing the environmental footprint of dairy farming, contributing to more sustainable and environmentally responsible farming practices.

## 1. Introduction

Global population growth, urbanisation, and changing dietary habits are increasing food demand [1], while climate change poses threats to agricultural sustainability through extreme weather and temperature [2]. These factors negatively influence food security and environmental protection [3]. Rising incomes and evolving consumer preferences are also increasing demand for animal products, highlighting the economic relevance of livestock production [4].

Livestock farming is a crucial source of food, especially in rural areas [5]. It occupies 26% of global land, contributing to increased resource use, deforestation, and pollutant emissions [6,7]. According to the European Environment Agency, agriculture accounted for approximately 11% of total greenhouse gas emissions in the European Union in 2021 [8].

Among pollutant gases, ammonia (NH_3_) is a concern for its role in eutrophication of water and soil. Additionally, NH_3_ contributes indirectly to global warming due to the formation of nitrous oxide (N_2_O) [9]. In livestock barns, NH_3_ is primarily realised by the urinary excretion of cattle, resulting in a significant loss of nitrogen in the atmosphere. Estimating NH_3_ emissions can be challenging due to the various factors that influence the estimation procedure. Multiple factors, including housing system and floor type, influence emission factors [10]. Facilities with fixed stalls showed NH_3_ emissions of 6.9 g LU^−1^ d^−1^, whereas free-stall cubicle systems accounted for 29.3 g LU^−1^ d^−1^. Regarding floor type, perforated floors resulted in emission factors of 33.3 g LU^−1^ d^−1^ compared to 27.6 g LU^−1^ d^−1^ for solid floors. Emissions were significantly influenced by temperature (*p* < 0.001). Furthermore, CH_4_ and NH_3_ concentrations showed a strong positive correlation (r = 0.80).

Another significant pollutant gas is methane (CH_4_), a well-known greenhouse gas (GHG) emitted from livestock facilities. Its release into the atmosphere contributes to increasing global warming. In dairy farming, CH_4_ is produced by manure and enteric fermentation of cows, accounting for a third of total livestock-related emissions. The mean global estimation of CH_4_ emissions is estimated to be 383.5 million tons per year [11].

In the literature, many research studies have analysed the influencing factors of NH_3_ and CH_4_ concentrations and emissions from naturally ventilated dairy barns. NH_3_ and CH_4_ emissions factors are based on several conditions, including housing system (i.e., cubicle free-stall or loose barn); waste management practices [12]; ventilation systems and cooling systems [13,14]; diet [15,16,17]; micro-climatic conditions in the barn (e.g., temperature, relative humidity, and ventilation rate) [18,19]; animal diet and nitrogen losses during digestion [20]; animal activity, such as standing, lying, and feeding [21,22]; and production demands related to milk yield, livestock growth, and reproduction [23]. In this context, the influence of animal activity on NH_3_ and CH_4_ concentrations and emissions has been analysed in a limited number of studies.

Recently, an artificial neural network (ANN) has been applied in the scientific literature to estimate concentrations and emissions related to the livestock sector [24,25]. A neural network is a computational model inspired by the structure and functioning of the human brain. It consists of interconnected nodes, or artificial neurons, organised into layers [26]. The network receives data input, processes it through hidden layers by using weighted connections, and produces an output [27]. Neural networks are trained by using labelled datasets, adjusting the weights between neurons through iterative learning processes. In the literature, some studies have applied ANN to predict NH_3_ and CH_4_ concentrations and emissions from livestock farming, as highlighted in a recent review [28].

In a recent study, authors estimated NH_3_ emissions by applying the mass balance method in manure storage of a dairy farm by training and testing ANN models based on weather variables, manure characteristic, and number of animals [29]. The recent investigation carried out in [30] predicted nitrogen excretion from indoor dairy cattle; in emission estimation, dietary variables, livestock variables, and excreta characteristics were considered as input [30].

A study investigated the NH_3_ emissions from an indoor loose stall dairy barn by using statistics and machine learning approaches. Data was collected in a naturally ventilated barn, and implied estimations of hourly emission values were derived from the ventilation rate, time, temperature, wind speed and direction [31].

These studies lack a systematic evaluation of the most relevant input variables for predicting concentrations and emissions. In detail, animal activity has not been considered as an input variable in any of the studies aiming to carry out emission prediction. Generally, models incorporating animal activity and diet often lack environmental variables and prove to have accuracy loss [32]. Therefore, there is a need for further investigations to analyse the influence of input variables on NH_3_ and CH_4_ concentrations and emissions. In fact, to the authors’ knowledge, the application of an ANN in this context has not been analysed yet. Therefore, this study aimed to evaluate how climatic conditions, animal activity, and diet intake characteristic of the Mediterranean area influence the prediction of NH_3_ and CH_4_ concentrations and emissions from dairy cattle housing. Based on these assumptions, the objective of this study was to compare ANN models based on different sets of input variables to identify optimal combinations for accurately predicting representative gas concentrations and emission rates in Mediterranean dairy barns.

## 2. Materials and Methods

### 2.1. Barn Features and Feed Composition

**Figure 1:**
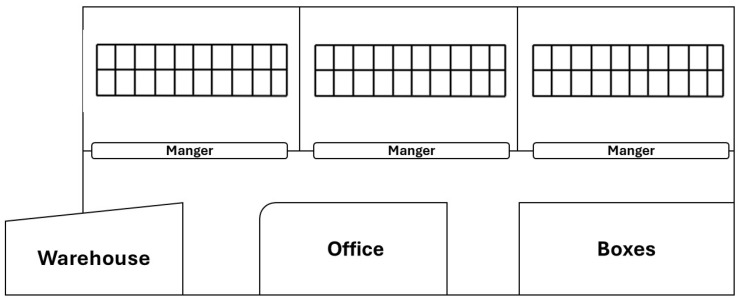
Planimetry of the barn under study. The barn hosted an average of 56 Frisian cows, arranged in three separated pens. Cleaning operations were conducted once a day at approximately 07:30 a.m. by using a mechanical tractor with a scraper. Cows were milked twice a day, at around about 6:00 a.m. and 6:00 p.m. in a separate milking parlour, while feed was delivered at 12 a.m.

Data were collected in a cubicle free-stall dairy barn (Figure 1) located in Vittoria (37°01′ N, 14°32′ E), (RG), Italy, 234 m asl. The local climate in Vittoria (RG) is classified as Mediterranean (Csa in the Köppen classification), with a yearly average air temperature of 18.90 °C and 79.87% relative air humidity. The naturally ventilated barn structure was made of steel and concrete with a ridge vent located 7 m above the ground. The dimensions measured approximately 55.50 m in length and 20.80 m in width. It presented three completely open sides (SE, NE, and NW). The SW side was enclosed by a wall containing four small openings.

The investigation period was from 1 November 2021 to 30 November 2021.

During the study period, the cows’ diet consisted of a mixed ratio with concentrate. The diet, delivered once a day, was a unifeed composed of alfalfa hay, hay, grass hay, beet, urea, milk performer 170 A1 produced by Purina©, St. Louis, MO, USA (with the following ingredients: 20.5% of crude protein, 4.85% of crude fat, 8.31% of crude fibre, 5.99% of crude ash, and 0.25% of sodium), citrus pulp, fat, yeast, flaked maize, flaked soybeans, cottonseed, salt, and sodium bicarbonate. Table 1 shows the feed intake in the barn when the quantity, expressed in kilograms, changed due to animal or management needs.

### 2.2. Environmental Variables and Gas Concentration Measurements

Climate and micro-climatic variables were recorded by using two weather stations (MeteoSense 2.0 GPRS + LAN, Netsens, Florence, Italy), installed inside and outside the barn. Each station was equipped with an ultrasonic anemometer and a thermo-hygrometer. The outdoor station measured air temperature, relative humidity, and wind speed and direction, with a resolution of 0.01 m s^−1^, an accuracy of ±0.15 °C for temperature, and a sensitivity of 0.04% RH/°C and a precision of 2% (at 20 °C) for humidity. The indoor station was positioned 2.20 m above the central pen within the barn to monitor internal environmental conditions. Both stations acquired climatic and micro-climatic variables continuously throughout the experimental period.

CH_4_, NH_3_ and CO_2_ concentrations were continuously measured using an INNOVA photoacoustic gas analyser, consisting of a Multigas Monitor model 1412i and a multipoint sampler 1409/12 (Lumasense Technology A/S, Kleyerstr, Frankfurt/Main, Germany). Six sampling points (SLs) were positioned indoor the barn along the feeding alley, with two SLs at three different heights from the floor (i.e., 0.4 m, 1.5 m and 2.7 m from the floor). Another outdoor SL was located 8 m above the roof to measure background concentrations. The INNOVA analyser was calibrated just before the measurements. The setup of the INNOVA had a sample integration time of 5 s. The detection limit of the Multi-Gas Analyser was 0.2 ppm for NH_3_, 0.4 ppm for CH_4_, and 1.5 ppm for CO_2_.

### 2.3. Recording of Animals’ Activities 

Animal activity was continuously monitored by using a 24 h video recording system consisting of ten cameras (Kon.Li.Cor, Perugia, Italy) installed at a height of 4 m above the floor in the central pen. The analysis of cows’ activity from the video recordings was performed by a trained operator. Visual assessments were carried out every 15 min to record the number of cows engaged in various activities (e.g., feeding, standing, walking, or lying). These observations were then used to calculate behavioural indices, namely the Cow Lying Index (CLI) and Cow Activity Index (CAI), as described in previous studies [33,34].

### 2.4. Emission Calculation Using CO_2_ Mass Balance Method

NH_3_ and CH_4_ emissions were estimated by using the CO_2_ mass balance method. The barn ventilation rate *Q* (m^3^ h^−1^) was calculated as

$$Q=\frac{P_{CO2}\times N}{\left(C_{CO2in}-C_{CO2out}\right)}$$

where *P*_CO2_ is the CO_2_ excretion rate per cow (g cow^−1^ h^−1^), *N* is the number of cows housed in the barn, and *C*_CO2in_ and *C*_CO2out_ are the average hourly concentrations of CO_2_ inside and outside the barn, respectively (g m^−3^). In this approach, the CO_2_ excretion rate depends on animal heat production. Since, in barns without deep litter, the CO_2_ produced from manure represents less than 4% of the total production [33], the excretion rate was calculated as follows [34,35,36]:

$$q_{t}=5.6\times \;m^{0.75}+1.6\times 10^{-5}\times p^{3}+22\times y$$

$$CF=4\times 10^{-5}\times {\left(20-T_{i}\right)}^{3}+1$$

$$q_{cor}=q_{t}\times CF$$

$$P_{CO2}=0.299\times q_{cor}$$

where $q_{t}$ is the total heat production (W), m is the average animal mass (kg cow^−1^), p is the number of days after insemination (d), y is the milk yield (kg d^−1^), CF is the temperature correction factor, $T_{i}$ is the indoor air temperature (°C), and $q_{cor}$ is the corrected total heat production (W).

The emission rate of NH_3_ and CH_4_ was then computed as

$$E_{t}=Q\times \left(C_{in}-C_{out}\right)$$

where $E_{t}$ is the gas emission rate (g h^−1^), Q is the ventilation rate obtained from the CO_2_ balance (m^3^ h^−1^), and $C_{in}$ and $C_{out}\;$ are the indoor (average of the four sampling locations) and outdoor concentrations (g m^−3^), respectively. To enable comparison with other studies, the emissions were expressed per livestock unit (LU), where one LU corresponds to a 500 kg animal mass. The emission rate per LU was determined as

$$E=\frac{E_{t}\times LU}{N\times m}$$

### 2.5. Artificial Neural Network Models and Development Method

The ANN models used in this study were created in MATLAB© by MathWorks. All experimentations were performed in a computer running Microsoft Windows 11 Home© ×64 bit with an Intel(R) Celeron(R) N4020 CPU 1.10 GHz, RAM 4.00 GB, Intel(R) UHD Graphics 600, manufactured by ASUSTeK Computer Inc. (Taipei, Taiwan). MATLAB software versions R2025a and R2025b were used for model development and testing.

The following subsections describe in detail the dataset definitions, the model architecture, the training process, and the performance evaluation.

#### 2.5.1. Dataset Definition

The dataset consisted of 718 records and included the following variables: hour of the day, indoor and outdoor air temperatures, indoor and outdoor air relative humidities, indoor and outdoor wind directions, indoor and outdoor wind speeds, CLI, CAI, and diet composition.

All variables were acquired during the measurement campaign, and the mean hourly value of each variable was considered for this study. For the prediction of NH_3_ emissions, the dataset also included indoor and outdoor NH_3_ and CO_2_ concentrations, while indoor and outdoor CO_2_ and CH_4_ concentrations were used for the prediction of CH_4_ emissions.

#### 2.5.2. Model Architecture, Training Process and Evaluation Metrics

**Figure 2:**
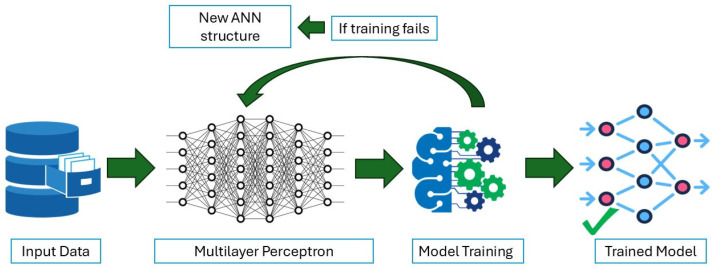
Flowchart of the rationale applied in this study to obtain the ANN models.

In this study, the optimal ANN model was determined by following a rational, incremental approach. The process started with the simplest structure, consisting of 1 hidden layer with 5 neurons. When the configuration failed to achieve the desired accuracy, an additional hidden layer was added, containing half of the neurons of the preceding hidden layer, as described in [24] and show in the flowchart below (Figure 2).

The model obtained, a Multilayer Perceptron (MLP) with a 20–10 structure, was used for all tests. Model performance was evaluated by using five evaluation metrics: the coefficient of determination (R^2^), coefficient of correlation (R), Mean Absolute Error (MAE), Root Mean Square Error (RMSE), Standard Deviation (SD) and Mean Square Error (MSE). Models were considered satisfactory when R^2^ was above 0.70 and R was above 0.80. These performances were evaluated through linear regression analysis between target values and network outputs using MATLAB’s built-in regression function.

Optimal network performance is indicated by an R value equal to 1 (i.e., perfect correlation), with an R^2^ value equal to 1 representing perfect agreement between predicted and observed values. Satisfactory network performance is indicated by an R value above 0.80 and an R^2^ value above 0.70.

It is relevant to highlight that although the MSE, RMSE, SD and MAE do not have predefined thresholds, they remain valuable indicators for evaluating model performance [37].

Further tests were performed by adding more neurons to the layers, yet no significant accuracy improvement was observed.

The training was carried out by using default MATLAB© variables and hypervariables, as summarised in Table 2. To prevent overfitting, an early stopping approach was applied, whereby training was interrupted once performance loss occurred during validation checks.

#### 2.5.3. Performance Comparison of ANN Models for Predicting NH_3_ and CH_4_ Concentrations and Emissions 

The optimal combination of input variables (climatic variables, animal activity and diet) was analysed to predict NH_3_ and CH_4_ concentrations and emissions. A series of prediction tests was carried out by using several configurations for concentration and emission predictions, as shown in Table 3 and Table 4, respectively. ANN model performance was evaluated through the comparison of evaluation metrics for each test. To evaluate the best-performing ANN models, linear regression analysis and prediction plots were applied. ANN models associated with variables that did not meet the quality thresholds were excluded from further analysis.

## 3. Results

In accordance with the methodology described in Section 2, a screening of various models was conducted to identify the best performing model. The ANN structures were described by using a numerical notation (e.g., 20–10), representing the number of neurons in the sequential hidden layers. Among the tested configurations, the MLP (20–10) model emerged as the most suitable structure, achieving the highest prediction accuracy. Consequently, this specific configuration was selected for both the NH_3_ and CH_4_ concentration and emission prediction tasks. The same ANN architecture (20–10) allowed a comparison of predictive performance across configurations.

### 3.1. Concentrations Outcomes

Table 5 summarises the model performance results for the prediction of NH_3_ and CH_4_ concentrations by using different combinations of input variables, including climatic variables, animal activity, and diet.

For NH_3_ concentrations, models based on single input categories showed moderate performance, with climatic variables providing the highest accuracy (R = 0.89; R^2^ = 0.78), followed by activity and diet. Model performance improved when multiple input variables were combined, particularly when climatic variables and diet were used as input (R = 0.92; R^2^ = 0.84). The highest predictive accuracy was obtained when climatic variables, animal activity, and diet were jointly included (R = 0.93 and R^2^ = 0.85) with the lowest error values.

For CH_4_ concentrations, climatic variables alone yielded higher accuracy than models obtained with animal activity or diet input variables. The inclusion of additional input variables improved model performance, particularly when climatic variables and diet were applied (R = 0.92 and R^2^ = 0.83). The best overall results were observed when all three variable groups were included (R = 0.96 and R^2^ = 0.89).

Variables that failed to meet quality requirements were discarded, and the corresponding ANN models were excluded in the subsequent linear regressions and plot analyses. Notably, while CH_4_ predictions satisfied the required quality thresholds, their prediction errors were higher than those for NH_3_.

**Figure 3:**
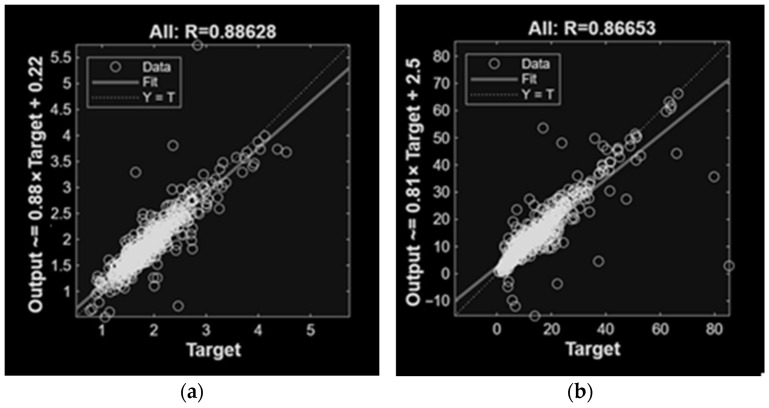
Linear regression of MLP models involved in NH_3_ (**a**) and CH_4_ (**b**) predictions of the concentrations with only climatic variables as inputs.

**Figure 4:**
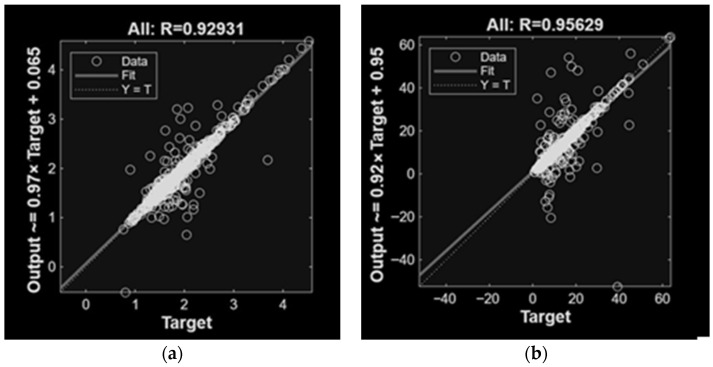
Linear regression of MLP models involved in NH_3_ (**a**) and CH_4_ (**b**) predictions of the concentrations with all variables as inputs.

Figure 3 and Figure 4 present the linear regression results for the best ANN models trained with different variables. Results demonstrated good agreement between the regression fit and the ideal Y = T line, confirming the prediction capability of the proposed models. As expected, the MLP models succeeded in making accurate predictions, effectively capturing the relationships between input variables and concentrations. Notably, prediction including climatic variables achieved the best results.

**Figure 5:**
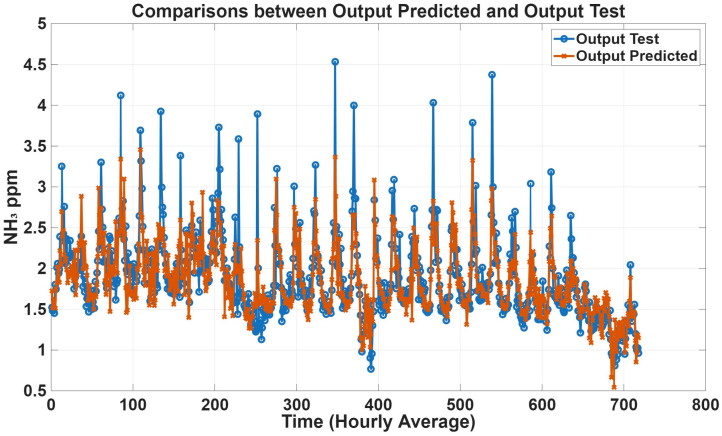
Comparison plots of predicted and measured NH_3_ concentrations with only climatic variables as inputs. The predicted line (orange) presents statistical metrics (R = 0.89, R^2^ = 0.78, and RMSE = 0.24).

**Figure 6:**
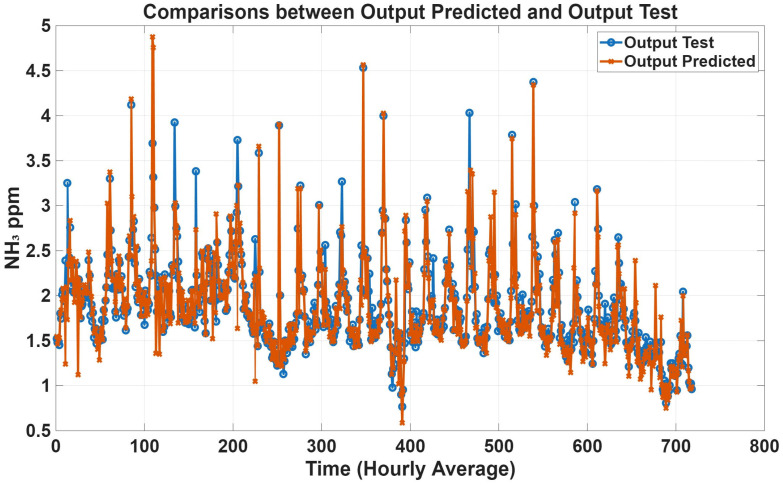
Comparison plots of predicted and measured NH_3_ concentrations with all variables as inputs. The predicted line (orange) presents statistical metrics (R = 0.93, R^2^ = 0.85, and RMSE = 0.19).

**Figure 7:**
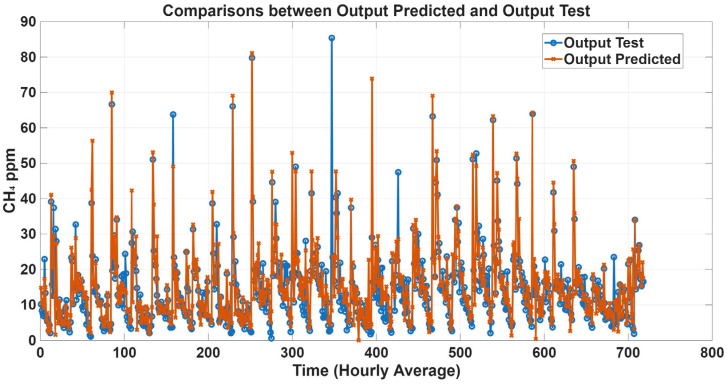
Comparison plots of predicted and measured CH_4_ concentrations with only climatic variables as inputs. The predicted line (orange) presents statistical metrics (R = 0.87, R^2^ = 0.74, and RMSE = 5.44).

**Figure 8:**
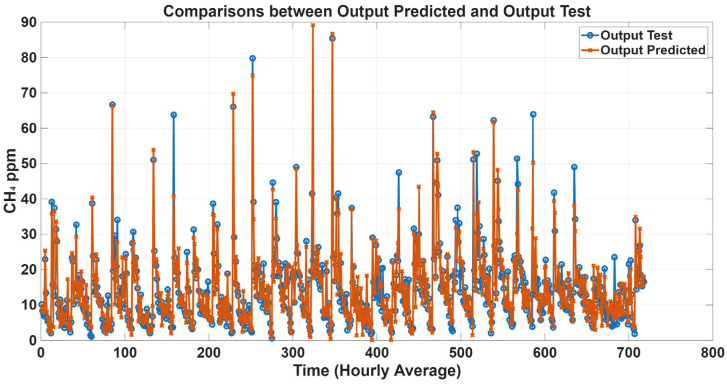
Comparison plots of predicted and measured CH_4_ concentrations with all variables as inputs. The predicted line (orange) presents statistical metrics (R = 0.96, R^2^ = 0.89, and RMSE = 4.50).

To further evaluate model performance, time-series predictions plots were generated based on historical concentration data collected in the barn. Figure 5, Figure 6, Figure 7 and Figure 8 show a comparison between predicted and observed concentrations, with each prediction paired to its corresponding measured value. Overall, the predicted values showed good agreement with the measured data, confirming the reliability of the proposed models.

### 3.2. Emissions Outcomes

Table 6 presents the model performance results for NH_3_ and CH_4_ emissions.

For NH_3_ emissions, models using single input categories showed variable performance. Gas concentrations alone provided higher accuracy (R = 0.91; R^2^ = 0.82) than when climatic, activity, or dietary variables were used individually. The inclusion of concentrations with additional inputs improved model performance, particularly when combined with climatic variables (R = 0.94; R^2^ = 0.89). The highest predictive accuracy was achieved when concentrations, climatic variables, animal activity, and diet were jointly included, including R = 0.96 and R^2^ = 0.92 with the lowest error values.

For CH_4_ emissions, models based on concentrations alone also showed good performance (R = 0.93; R^2^ = 0.86), while models relying solely on climatic, activity, or dietary variables resulted in lower accuracy. When concentrations were integrated with other variables as inputs, the prediction performance improved. Specifically, concentrations and diet as input variables reached R = 0.96 and R^2^ = 0.93. The best overall results were obtained when all input variables were included, achieving R = 0.97 and R^2^ = 0.92 and the lowest error metrics.

**Figure 9:**
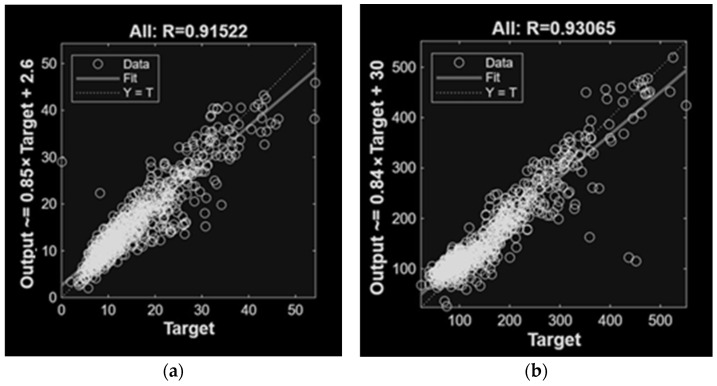
Linear regression of MLP models involved in NH_3_ (**a**) and CH_4_ (**b**) predictions of emissions, with concentration variables as inputs.

**Figure 10:**
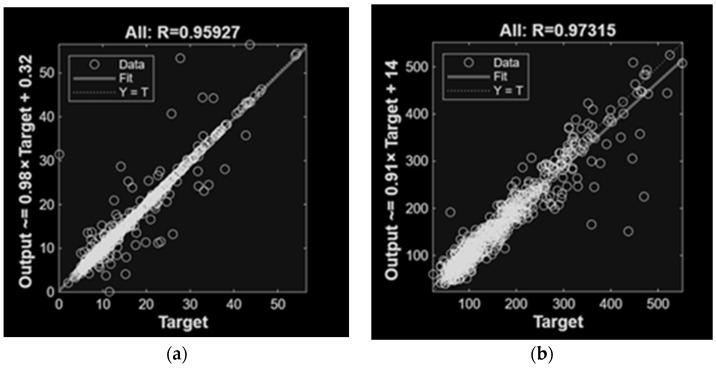
Linear regression of MLP models involved in NH_3_ (**a**) and CH_4_ (**b**) predictions of emissions, with concentration, climatic, animal activity and diet variables as inputs.

While CH_4_ models satisfied the required quality criteria, their prediction errors remained higher than those obtained for NH_3_. Figure 9 and Figure 10 present the regression results for the top-performing ANN models trained with different input variable configurations. The results demonstrate strong agreement between the regression fit and the ideal Y = T line, confirming the predictive capability of the proposed models. As expected, the MLP models achieved accurate predictions, effectively capturing the relationships between input variables and the emissions. Notably, incorporating gas concentration variables obtained the best performance.

**Figure 11:**
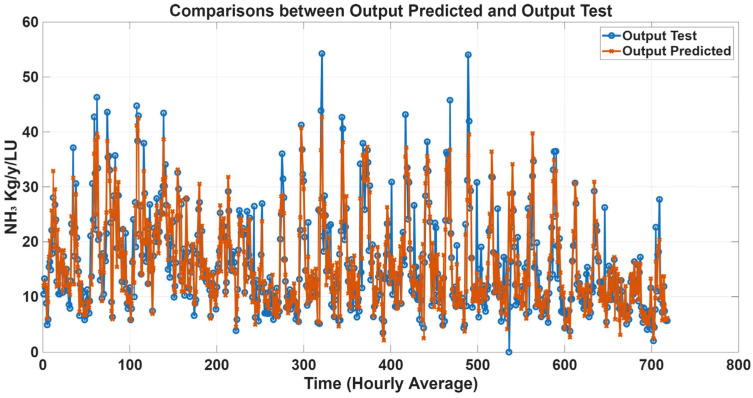
Comparison plots of predicted and estimated NH_3_ emissions with gas concentration variables as inputs. The predicted line (orange) presents statistical metrics (R = 0.91, R^2^ = 0.82, and RMSE = 3.57).

**Figure 12:**
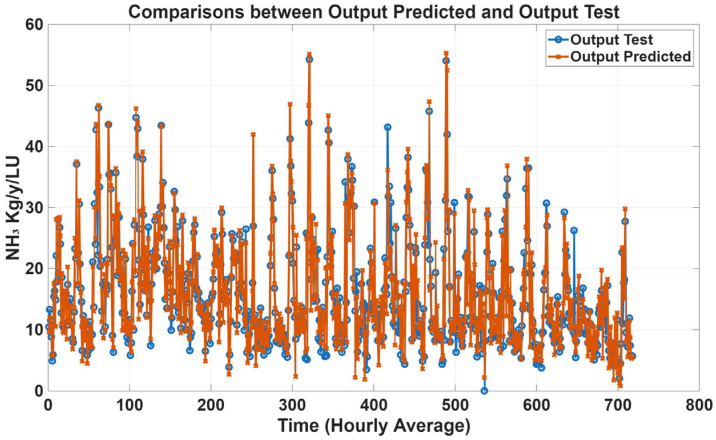
Comparison plots of predicted and estimated NH_3_ emissions with all variables as inputs. The predicted line (orange) presents statistical metrics (R = 0.96, R^2^ = 0.92, and RMSE = 2.56).

**Figure 13:**
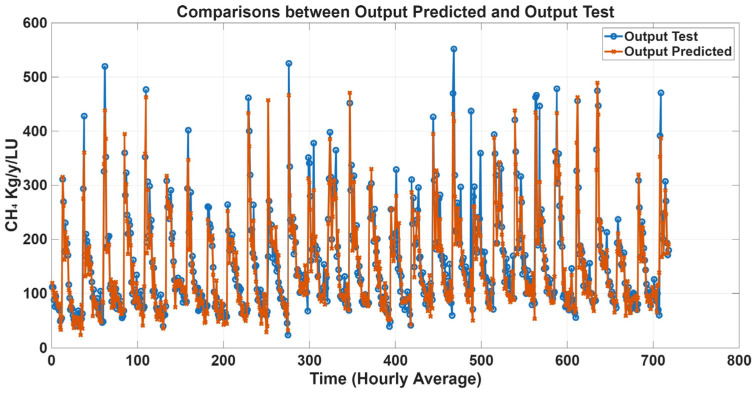
Comparison plots of predicted and estimated CH_4_ emission with gas concentration variables as inputs. The predicted line (orange) presents statistical metrics (R = 0.93, R^2^ = 0.86, and RMSE = 34.50).

**Figure 14:**
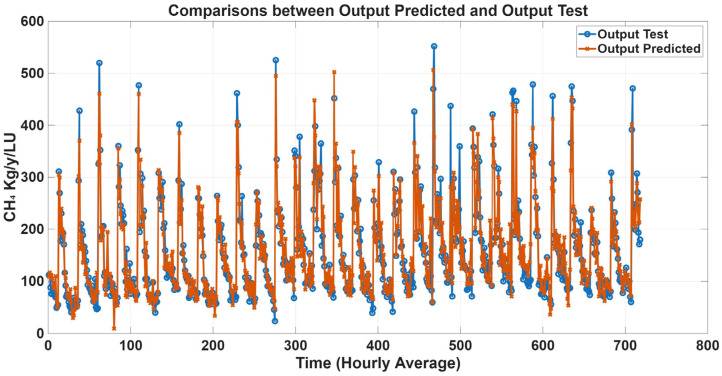
Comparison plots of predicted and estimated CH_4_ emission with all variables as inputs. The predicted line (orange) presents statistical metrics (R = 0.97, R^2^ = 0.92, and RMSE = 21.68).

Time-series prediction plots were generated to further assess model performance using estimated emission data. Figure 11, Figure 12, Figure 13 and Figure 14 compare predicted and estimated emissions over time, with each prediction paired to its corresponding measured value. These plots provided a comprehensive visualisation of model accuracy, enabling evaluation of both absolute prediction errors and the ability of the models to capture temporal emission dynamics, including daily pattern and responses to changes in environmental conditions, animal activity, and dietary intake. Visual inspection revealed that predicted values closely tracked measured emissions, accurately reproducing peak events and baseline levels. The strong temporal agreement between predictions and observations confirmed the reliability and robustness of the proposed MLP models.

## 4. Discussion

This study evaluated the capability of prediction models to accurately forecast gas concentrations and emissions in a Mediterranean dairy barn, incorporating climatic, dietary, activity, and gaseous concentration variables as inputs. The results demonstrate that ANN models can predict NH_3_ and CH_4_ concentrations and emissions, showing good agreement between measured or estimated data and predicted outcomes. Overall, the results confirm that ANN models incorporating a comprehensive set of climatic, activity, and dietary variables provide better predictive accuracy for both NH_3_ and CH_4_ concentrations and emissions. This highlights the importance of integrated, multi-source input strategies in livestock emission modelling.

The results of this study align with previous publications involving predicting concentrations and emissions in the livestock sector [24,29,31]. The investigation presented in [29] applied ANN models to predict NH_3_ emissions from dairy manure storage, using manure characteristics, temperature and NH_3_ concentrations as input variables. The outcomes demonstrated how ANN models generalise and predict NH_3_ emissions with good agreement, involving environmental, manure, and livestock inputs. Although the aim of the article was comparable to that of this study, direct comparison is limited due to differences in evaluation metrics, emission estimation output and input variable selection [29]. Similarly, in [38] an ANN model was developed to forecast NH_3_ emissions from piggeries, incorporating NH_3_, CO_2_, and H_2_O concentrations along with indoor and outdoor climatic variables. However, the reported accuracy was lower than that achieved in this study, and training algorithms applied during the training of the ANN models were not disclosed, limiting reproducibility.

Regarding CH_4_ predictions, the application of ANN for predicting enteric CH_4_ was investigated in [39], incorporating animal activity, climatic variables, milk characteristics and management practices. Although their study closely aligns with the objectives of this research, the reported prediction accuracy was lower compared to the results obtained in this study. Similarly, in [40], CH_4_ emissions were forecasted by developing several ANNs and statistical models, using animal and milk nutrient variables as input variables. They found that ANN models performed better than the applied statistical model. However, the reported RMSE (87.28 to 99.39) values were higher than those obtained in the present study.

Environmental variables have a significant influence on emission modelling and have been extensively investigated in the literature. These variables often show complex, nonlinear interactions influenced by climatic conditions requiring ANN models to adapt their weights to capture such relationship [41,42]. However, collecting reliable data remains challenging due to the high cost of the devices, the complexity of gas dynamics, and the relations between pollutants and barn conditions [43,44].

In this study, environmental variables were monitored by using accurate devices that require significant economic efforts not only for instrumentation but also for maintenance. The identification of the optimal ANN with specific variables allowed the definition of a specific methodology based on accurate data. Further analysis could focus on improving monitoring by using low-cost and user-friendly devices, but such solutions are often not affordable or sufficiently reliable for widespread application. Despite these challenges associated with data quality and sensor deployment, the results indicate that ANN models can reduce the cost of computational analysis and improve the efficiency of time-dependent analysis [45].

Traditional statistical approaches have been applied in the scientific literature to estimate pollutant gas emissions, such as CH_4_, CO_2_, N_2_O, and NH_3_, by using linear regression, multivariate analyses, and cross-over trial designs [46,47,48,49]. These methods have demonstrated the feasibility of mathematical equations for emission estimation and analysis. Comparative studies have also assessed statistical and ANN-based approaches for NH_3_ emission prediction [31]. However, statistical approaches in emission forecasting have been outperformed by ANN-based methods owing to improved predictive performance and enhanced understanding of barn dynamics [24,50,51,52]. The results of this study demonstrate the effectiveness of ANN models in predicting emissions from dairy housing systems using inputs related to climatic conditions and barn management (i.e., feed, animal activity). Although few studies [21,39] have explored the influence of animal activity and dietary on concentrations and emissions, the novelty of this study introduces their combined integration with climatic variables in an ANN-based approach to improve prediction accuracy for both NH_3_ and CH_4_ concentrations and emissions.

In recent years, ANN applications to livestock production have marked an important advancement in agricultural practices. These systems offer enhanced insights into animal activity, health, and overall well-being [53]. ANNs can process vast datasets from sensors and monitoring devices, tracking variables such as feeding patterns, movement, and physiological indicators. This real-time analysis enables farmers to detect anomalies promptly, enhancing disease prevention and livestock management [54]. Furthermore, ANNs contribute to precision livestock farming (PLF) by optimising feed formulations, reducing waste, and improving resource efficiency [55]. This integration of technology and agriculture not only enhances productivity but also promotes sustainable practices [56]. As the agricultural landscape evolves to meet the demands of a growing global population, the integration of predictive models into livestock production emerges as a key driver for efficient, humanitarian, and economically viable farming practices [57].

In the context of PLF, livestock activity has been investigated by using different devices and approaches, including GPS, video surveillance, accelerometers, and tags [58,59,60,61], with the aim of enhancing animal health and welfare. In the past decade, there has been a growing interest in the application of deep learning (DL) models useful for studying livestock activity in relation to animal health issues. Main monitoring systems include (i) computer vision-based systems that use camera technologies and deep learning algorithms for automatic activity monitoring; (ii) DL models used for activity recognition and classification; (iii) advanced neural networks for image analysis; and (iv) wearable sensors and devices for tracking movement, body temperature and physiological variables [62].

These PLF technologies generate large volumes of data that require advanced analytical tools for interpretation. In this context, the integration of ANNs with PLF systems represents a promising approach for comprehensive livestock management. ANNs have proven instrumental in several applications, including animal facial recognition for individual identification and tracking [37], movement tracking for improved understanding of animal activity and migration patterns [63,64], and activity classification for categorising animal responses to various stimuli [65,66]. Notably, low-cost devices combined with neural networks offer cost-effective solutions for activity monitoring [67]. Despite these advances, recent ANN applications have not yet fully incorporated animal activity and dietary variables for predicting gaseous emissions from livestock buildings. In this context, proposed MLP neural networks offer a promising approach for integrating PLF-derived behavioural data with environmental and nutritional variables to predict NH_3_ and CH_4_ emissions from dairy facilities. This integration enables a more comprehensive understanding of emission dynamics by capturing the combined effects of climatic conditions, animal behaviour, and feeding management on barn air quality.

## 5. Conclusions

The findings demonstrate that ANN models incorporating animal activity, diet and environmental variables represent effective tools for concentration and emission prediction from a Mediterranean dairy barn, contributing to improved environmental management and sustainable dairy farming practices. The novelty of this research study lies in three key aspects. Firstly, it provided an analysis of ANN model design, including architecture, hypervariables, and preprocessing techniques. Secondly, the integration of animal activity variables along with environmental and dietary variables addressed a gap identified in previous research, where some information on either activity or environmental variables was often omitted. Thirdly, the models were developed and validated under Mediterranean climatic conditions, which have not been investigated in depth in the literature.

Despite promising results, limitations related to dataset size and random data partitioning suggest the need for further validation. Future research should focus on testing these models across larger datasets, diverse climatic regions, and multiple seasons to enhance generalisability and robustness. Ultimately, the adoption of such predictive models contributes to more sustainable and efficient environmental management in the livestock sector.

## Figures and Tables

**Table 1:** Dietary composition and quantities during the investigation. Ingredients are reported in kg.

Day	Alfalfa Hay	Hay	Grass Hay	Beet	Urea	170 A1	Citrus Pulp	Fat	Yeast	Flaked Maize and Soybean	Cottonseed	Salt	Bicarbonate
1	4.00	3.25	3.00	1.00	0.02	10.00	21.00	0.20	0.05	4.10	1.50	0.10	0.20
11	4.00	3.25	3.00	1.00	0.02	10.50	23.00	0.20	0.05	4.60	1.50	0.10	0.20
18	3.50	3.25	3.00	1.00	0.02	11.00	27.00	0.20	0.05	5.10	1.50	0.10	0.20
20	3.50	3.25	4.60	1.00	0.02	11.00	27.00	0.20	0.05	5.10	1.50	0.10	0.20
22	3.50	3.25	5.20	1.00	0.02	11.00	27.00	0.20	0.05	5.10	1.50	0.10	0.20
26	3.50	3.25	5.50	1.00	0.02	11.00	27.00	0.20	0.05	5.10	1.50	0.10	0.20
30	3.50	3.25	2.50	1.00	0.02	11.00	27.00	0.20	0.05	5.10	1.50	0.10	0.20

**Table 2:** Settings of the ANN model applied, with default values reported as provided by MATLAB.

Variable/Hypervariable	Default Value
Transfer function input	tansig
Transfer function output	purelin
Data normalisation (input and output)	min/max
Training ratio (data, test, and validation)	70:15:15
Training algorithm	Levenberg–Marquardt
Epochs	1000
L2 regulation	not implemented

**Table 3:** ANN variables for predictions of gas concentrations.

Variables	Output
Climatic variables	Concentrations
Animal activity	Concentrations
Diet	Concentrations
Climatic variables and animal activity	Concentrations
Climatic variables and diet	Concentrations
Diet and animal activity	Concentrations
Climatic variables, animal activity and diet	Concentrations

**Table 4:** ANN variables for predictions of gas emissions.

Variables	Output
Concentrations	Emissions
Climatic variables	Emissions
Animal activity	Emissions
Diet	Emissions
Concentrations and climatic variables	Emissions
Concentrations and animal activity	Emissions
Concentrations and diet	Emissions
Climatic variables and animal activity	Emissions
Climatic variables and diet	Emissions
Animal activity and diet	Emissions
Concentrations, climatic variables and animal activity	Emissions
Concentrations, climatic variables and diet	Emissions
Concentrations, animal activity and diet	Emissions
Climatic variables, animal activity and diet	Emissions
Concentrations, climatic variables, animal activity and diet	Emissions

**Table 5:** Results of the ANN model for concentration predictions. Variables and quality metrics were reported accordingly. Colours indicate the accuracy and the error of the model, from dark orange to bright green. Dark orange represents the worst outcome; bright green represents the best outcome.

Variables	NH_3_ in Concentrations							CH_4_ in Concentrations					
	ANN Structure	Accuracy Measures						Accuracy Measures					
		R	R^2^	MSE	RMSE	MAE	SD	R	R^2^	MSE	RMSE	MAE	SD
Climatic variables	20 10	0.89	0.78	0.06	0.24	0.15	0.24	0.87	0.74	29.65	5.44	2.38	5.44
Animal activity	20 10	0.81	0.64	0.10	0.31	0.21	0.31	0.64	0.42	68.00	8.24	5.50	8.24
Diet	20 10	0.77	0.59	0.11	0.33	0.22	0.33	0.53	0.28	83.92	9.16	5.95	9.16
Climatic variables and animal activity	20 10	0.91	0.81	0.05	0.22	0.10	0.22	0.89	0.78	25.68	5.06	2.24	5.06
Climatic variables and diet	20 10	0.92	0.84	0.04	0.20	0.11	0.20	0.92	0.83	18.97	4.35	2.21	4.35
Diet and animal activity	20 10	0.74	0.55	0.12	0.34	0.24	0.34	0.66	0.43	62.15	7.88	5.24	7.88
Climatic variables, animal activity and diet	20 10	0.93	0.85	0.04	0.19	0.09	0.19	0.96	0.89	20.34	4.50	2.01	4.50

**Table 6:** Results of the ANN model for emission predictions. Variables and quality metrics were reported accordingly. Colours indicate the accuracy and the error of the model, from dark orange to bright green. Dark orange represents the worst outcome; bright green represents the best outcome.

Variables	NH_3_ Emissions							CH_4_ Emissions					
	ANN Structure	Accuracy Measures						Accuracy Measures					
		R	R^2^	MSE	RMSE	MAE	SD	R	R^2^	MSE	RMSE	MAE	SD
Concentrations	20 10	0.91	0.82	12.75	3.57	2.71	3.57	0.93	0.86	1190.00	34.50	23.90	34.50
Climatic variables	20 10	0.64	0.41	46.27	6.80	5.02	6.80	0.81	0.65	3052.00	55.26	35.42	55.26
Animal activity	20 10	0.46	0.20	64.30	8.02	6.02	8.02	0.82	0.68	2792.00	52.84	35.54	52.84
Diet	20 10	0.69	0.47	41.51	6.44	4.66	6.44	0.79	0.63	3234.00	56.87	38.32	56.87
Concentrations and climatic variables	20 10	0.94	0.89	8.87	2.98	1.88	2.98	0.96	0.92	700.55	26.47	15.10	26.47
Concentrations and activity	20 10	0.91	0.83	13.00	3.61	2.47	3.61	0.94	0.88	1036.00	32.19	21.17	32.19
Concentrations and diet	20 10	0.92	0.85	11.95	3.46	2.40	3.46	0.96	0.93	621.10	24.92	15.10	24.62
Climatic variables and animal activity	20 10	0.78	0.60	30.90	5.56	4.05	5.56	0.79	0.62	3278.00	57.26	38.88	57.26
Climatic variables and diet	20 10	0.78	0.61	30.77	5.55	4.15	5.55	0.83	0.69	2668.00	51.66	33.21	51.66
Animal activity and diet	20 10	0.66	0.43	44.57	6.68	4.75	6.68	0.81	0.66	2720.00	52.15	34.31	52.15
Concentrations, climatic variables and animal activity	20 10	0.95	0.89	8.78	2.96	1.93	2.96	0.96	0.92	655.90	25.61	16.57	25.61
Concentrations, climatic variables and diet	20 10	0.92	0.85	11.84	3.44	2.38	3.44	0.95	0.91	813.28	28.52	20.56	28.52
Concentrations, animal activity and diet	20 10	0.94	0.89	8.96	3.00	2.02	3.00	0.95	0.90	830.61	28.82	16.77	28.82
Climatic variables, animal activity and diet	20 10	0.78	0.61	30.47	5.52	3.95	5.52	0.86	0.66	2915.00	54.00	14.76	54.00
Concentrations, climatic variables, animal activity and diet	20 10	0.96	0.92	6.56	2.56	0.85	2.56	0.97	0.92	470.24	21.68	12.87	21.68

## Data Availability

Data are available upon request to the corresponding author.

## Abbreviations

The following abbreviations are used in this manuscript:

NH_3_	Ammonia
ANN	Artificial Neural Network
PLF	Precision Livestock Farming
MLP	Multilayer Perceptron
CO_2_	Carbon Dioxide
CH_4_	Methane
N_2_O	Nitrous Oxide
SE	Southeast
NE	Northeast
NW	Northwest
SW	Southwest
RG	Ragusa
R^2^	Coefficient of Determination
R	Coefficient of Correlation
MAE	Mean Absolute Error
RMSE	Round Mean Square Error
MSE	Mean Standard Error
SD	Standard Deviation
